# Development of the function of autobiographical memories evoked by odor scale for older Japanese people

**DOI:** 10.3389/fpsyg.2022.945002

**Published:** 2022-07-22

**Authors:** Kohsuke Yamamoto, Kengo Yokomitsu, Takefumi Kobayashi

**Affiliations:** ^1^Faculty of International Studies, Osaka Sangyo University, Osaka, Japan; ^2^School of Psychological Sciences, University of Human Environments, Okazaki, Japan; ^3^Faculty of Human Studies, Bunkyo Gakuin University, Tokyo, Japan

**Keywords:** autobiographical memories, odor cue, memory functions, older people, aging

## Abstract

Odor-evoked autobiographical memory and related psychological changes have been evaluated based on several factors, such as emotionality, clarity, and re-experience. We developed the Function of Autobiographical Memories Evoked by Odor Scale (FAMOS) for older Japanese people as a new method for comprehensively evaluating the functions of odor-evoked autobiographical memory. We used the diary method; participants were instructed to record the contents of everyday involuntary autobiographical memories triggered by odor and complete the FAMOS. In Study 1, 600 older adults were surveyed to select items for the FAMOS and examine the factor structure. An exploratory factor analysis with PROMAX rotation using the maximum likelihood method resulted in four factors: (1) Evoking positive emotion, (2) Identity, (3) Facilitating communication, and (4) Coping with negative emotion. Sufficient reliability was demonstrated. In Study 2, the FAMOS's validity was examined in 600 older adults. We found significant correlations (*Pearson*) with the affective valence of odors, the Odor-evoked Autobiographical Memory Questionnaire, and other scales, confirming the validity of the FAMOS. In Study 3, the FAMOS was administered to 600 younger and 600 older adults; generational differences were compared for further validity. Older adults had higher “Evoking positive emotion,” “Identity,” and “Facilitating communication” scores on the FAMOS than younger adults, suggesting a fair degree of reliability and validity of the FAMOS.

## Introduction

The belief that odors are particularly evocative reminders of past experiences can be traced to a literary anecdote described by Proust (1960), in which the narrator was vividly reminded of his childhood upon dipping a madeleine biscuit into his tea. The so-called Proust phenomenon has been investigated in the field of psychology for over 30 years (Chu and Downes, 2000b; Larsson and Willander, 2009; Larsson et al., 2014; Saive et al., 2014; Hackländer et al., 2019). Several empirical studies have been published on the topic of odor-cued autobiographical memory (Herz and Cupchik, 1992; Chu and Downes, 2000a, 2002; Herz and Schooler, 2002; Herz, 2004). Herz and Cupchik (1992), for example, reported that odor-evoked memories tend to be highly emotional, vivid, specific, rare, and relatively old. Odor-cued memories were rated as more pleasant than other cue types but were thought of and spoken of less often. Subsequent studies have found odor-evoked autobiographic memories to be more emotional, vivid, and associated with a stronger feeling of being brought back in time to the occurrence of the event, compared to memories evoked by verbal label or other modality cues (Chu and Downes, 2002; Herz and Schooler, 2002; Herz, 2004; Willander and Larsson, 2006, 2007).

Functional studies have been actively conducted in the autobiographical memory domain from the viewpoint of how memories are of meaning to us (Pillemer, 2009). Autobiographical memory has been theorized to be used for at least three functions: self, social, and directive (Bluck, 2003; Bluck et al., 2005). The self function of autobiographical memory serves to support the continuity and coherence of the self and to support the maintenance of a desirable self-image. Social function refers to aspects of autobiographical memory that help form and maintain interpersonal relationships and positively influence interpersonal relationships and communication. The directive function of autobiographical memory helps with making various judgments and direct actions and is thought to be used as a basis for judgment. To measure the functioning of autobiographical memory as described above, the Thinking About Life Experiences (TALE) scale was developed (Bluck and Alea, 2011) and has been widely used. In addition, recalling positive autobiographical memories has been reported to serve other emotional regulation functions to moderate negative mood (e.g., Erber and Erber, 1994; Smith and Petty, 1995).

Thus, far, there has been insufficient research on the function of odor-evoked autobiographical memories. However, previous research has suggested a unique function for olfaction in autobiographical memory. For example, the Odor-evoked Autobiographical Memory Questionnaire (OEAMQ), which was developed to measure the characteristics of autobiographical memories evoked by odors in Japan, identified emotion and nostalgia as factors that predict an association between autobiographical memory and emotion regulation functions (Yamamoto and Sugiyama, 2017). The study also identified other factors, such as future action, as predictors of an association with the orienting function. In a related study in Japan, the Functions of the Autobiographical Memory in Shikohin inventory (FAMS)—developed to measure the function of autobiographical memories recalled after consuming alcoholic drinks, tea, coffee, or tobacco—also confirmed the function of “coping with negative emotion” (Yamamoto and Yokomitsu, 2019). Previous studies have indicated that emotion is one of the hallmarks of odor-evoked autobiographical memories (e.g., Engen, 1982). According to Engen (1982), odors are so strongly connected to emotions that they are considered to be an emotional judgment in themselves. Odors are intense emotional inducers that have been used as an effective means of eliciting mood in the study of emotion (e.g., Herz, 2002). In addition, neuropsychological studies have shown a relationship between the olfactory cortex and the amygdala-hippocampal complex (Arshamian et al., 2013). The amygdala is involved in processing emotional experiences and emotional or general memory. Therefore, autobiographical memories evoked by odors are expected to possess emotional functions. However, none of these scales have been developed to measure the functions of autobiographical memory with odor cues.

The purpose of this study was to develop the Function of Autobiographical Memories Evoked by Odor Scale (FAMOS) for older Japanese people. Recently, there have been interesting findings regarding odor-induced autobiographical memory in older people (e.g., Chu and Downes, 2000b; Maylor et al., 2002; Willander and Larsson, 2006, 2007). Willander and Larsson (2006, 2007), for example, reported that older people could evoke autobiographical memory by odor, and that the memories were from earlier in life than those evoked by visual or verbal cues. Chu and Downes (2000b) reported similar results. Even among older people with Alzheimer's disease, odor can serve as a potent cue for autobiographical memory retrieval (e.g., El Haj et al., 2018; El Haj, 2022). Therefore, we expect that some functions of odor-cued autobiographical memory will play an important role in supporting the wellbeing of older adults.

Recently, there has been an increased interest in odor-evoked involuntary memory (Hackländer et al., 2019; El Haj, 2022). The diary method has been mainly used in involuntary memory research (e.g., Berntsen, 1996; Berntsen and Hall, 2004; Yamamoto and Yokomitsu, 2019). The diary method provides a way to collect many different functions of memory because it is used in everyday life situations, not in a laboratory setting. Yamamoto et al. (2016) used the diary method in a study examining the functions of autobiographical memories involuntarily remembered by odor cues. In that study, they asked participants to provide written descriptions of their thoughts after an autobiographical memory was recalled by odor in their daily lives. The responses were then categorized into various functions of odor-evoked autobiographical memory, including direction of action, change of emotion, and reinterpretation of memory. Given the advantages of the diary method and its use in prior related research, we employed this method to examine the functions of involuntary autobiographical memories evoked by odors in daily life.

To develop the scale, we collected items as extensively as possible based on previous functional studies. First, we searched the literature using the keyword “autobiographical memory” in CiNii-Research (https://cir.nii.ac.jp)—the largest literature database in Japan run by the National Institute of Informatics—and obtained 818 articles. Second, each article was reviewed by co-researchers to determine its relevance to the function of autobiographical memory. Finally, in addition to the OEAMQ, FAMS, and TALE, we drew on the Reminiscence Function Scale (RFS) developed by Webster (1993, 1997) and the Autobiographical Reasoning Scale (ARS) developed by Sato (2015), as well as other functions found in Yamamoto, Inomata and Tomitaka (2016) research. The RFS is a scale that measures the functions of typical reminiscence acts in older people and includes unique functions that are not covered by the above functional scales, such as boredom reduction. The ARS (Sato, 2015) measures autobiographical reasoning, which is a self-reflective thought or narrative about an individual's past (Habermas and Bluck, 2000). Autobiographical reasoning plays an important role in connecting the self with each aspect of life and in examining in greater detail the self-functions that link the past and present.

In Study 1, we developed the FAMOS and examined its factor structure. In Study 2, we examined the concomitant validity of the scale using several related scales. In Study 3, we examined the discriminant validity of this scale by comparing generational differences between younger and older adults.

## Study 1

### Methods

#### Participants

Participants were recruited by a Japanese research firm and were provided reward points in accordance with the research firm's regulations. The criteria for participants were as follows: aged 65 years or older and who self-reported as having normal olfactory abilities. In addition, recruitment was carried out as widely as possible from registered participants living in all parts of Japan to avoid regional bias. Study 1's participants were 600 healthy older people in Japan (300 female participants, mean age = 70 years, standard deviation [SD] = 4.55, range 65–92 years).

#### Questionnaire

We selected 94 relevant items from the scales employed in previous functional studies of autobiographical memory. In selecting the items, we standardized the expressions through discussion among the co-researchers. The items were selected from the following scales, taking into account overlap between the scales' items:

##### The odor-evoked autobiographical memory questionnaire

This questionnaire, developed by Yamamoto and Sugiyama (2017), measures odor-evoked autobiographical memory characteristics and certain functions. The questionnaire was evaluated and found to be reliable and valid. It comprises seven factors: emotion, retrospective recollection, clarity, time information, future action, perceptual experience, and nostalgia. This study selected 6 items related to function from the following factors: future action, retrospective recollection, and nostalgia (e.g., “As the event was recalled, some new ideas or thoughts have come to mind definitely”) and 1 item from the emotion factor.

##### The thinking about life experience scale

This scale, developed by Bluck and Alea (2011), measures the frequency of the use of autobiographical memory functions. The Japanese version of the TALE has a three-factor model consisting of self, social, and directive functions (Ochiai and Oguchi, 2013). Seven items were used in this study (e.g., “When I want to understand how I have changed from who I was before.”).

##### The function of the autobiographical memory evoked by Shikohin scale

This scale, developed by Yamamoto and Yokomitsu (2019), measures the function of autobiographical memories that are recalled when a Shikohin product (alcohol, tea, coffee, and tobacco) is consumed. The questionnaire was evaluated and found to be reliable and valid. It consists of four factors: evoking positive emotions, the direction of action, coping with negative emotions, and nostalgia. Thirty items were used in this study (e.g., “I felt comfortable.”).

##### Reminiscence function scale

This scale for older people, developed by Webster (1993, 1997), measures the frequency of the use of reminiscence functions. The Japanese version of the RFS has a six-factor model comprising self-growth, boredom reduction, death preparation, intimacy maintenance, bitterness revival, and communication (Takigawa and Yokomitsu, 2019). To examine generational differences, we excluded items that were specific to one generation (e.g., “I reminisce because it helps me prepare for my own death.”). Finally, this study selected 31 items related to function (e.g., “I reminisce to avoid repeating past mistakes at some later date.”).

##### Autobiographical reasoning scale

This scale was developed by Sato (2015) specifically for Japanese people and measures the frequency of use of autobiographical reasoning. The ARS has a six-factor model consisting of lessons and growth, rehearsal, past self, present self, importance, and turning point. The present study selected 11 items related to function (e.g., “I thought I hadn't changed from then to now.”).

For functions that were not included in the items of these scales, 8 items (e.g., “I relaxed.”) were selected from the results of the free descriptions of the functions of autobiographical memories evoked by odors (Yamamoto et al., 2016).

#### Procedure

Based on previous studies (Berntsen, 1996; Berntsen and Hall, 2004), the diary method was used. The following instructions were provided online:

“In our daily lives, when we smell a smell or fragrance, we may suddenly think of various things, even though we are not trying to think of them. In this survey, when you suddenly remember something when you smell something, you are asked to record the trigger and the contents as soon as possible. Please do not try to remember or think about it, but write it down when it suddenly reminds you of something in the past. Only events in which you were directly involved should be included. Please do not write about other events associated with the event you recalled or social events in which you were not directly involved.

Between the time you read this explanation and the due date, you will be asked to record the necessary information when you have this kind of “spontaneous recall” experience. You may have had similar experiences several times during this period, but you only need to record the first time.”

Participants were asked to access the URL distributed by the research firm from their device (e.g., smartphone) and enter a response if they recalled a past event unintentionally cued by a specific odor in their daily life. They recorded the contents of the involuntary autobiographical memories and completed the event scale. We instructed “We would like to ask you about the changes that occurred to you as a result of remembering the event and the reasons why you remembered the event. For what purpose did you remember the past? Alternatively, what purpose was achieved by recalling a particular memory? Please read the following items and answer to the extent that they apply to you.” A total of 94 items were rated on a 7-point scale (1 = strongly disagree, 7 = strongly agree). The first case that occurred within 2 weeks of the implementation period was described.

### Results and discussions

The total number of memory descriptions was 600 (100%). Examples of odor cues were “perfume,” “osmanthus,” and “roses.” Examples of autobiographical memories are “The scent of perfume that the woman I was dating at the time liked to wear reminded me of a day on a date with that woman” and “The smell of a baby reminded me of the time my first son was born.” All data analyses were conducted using SPSS ver. 27.

To assess the FAMOS factor structure, exploratory factor analysis using the maximum likelihood method PROMAX rotation was conducted. The factor analysis was repeated, and considering the trend of the scree plot, consistency with the previous results, and interpretability, it was determined that a four-factor solution with 17 items was appropriate after excluding items with factor loadings of <0.70. The results of these analyses and the mean, SD, and commonality of each factor are shown in Table 1. Factor 1, named “Evoking positive emotion,” comprised five items such as “I felt comfortable” and “I relaxed.” Factor 2, named “Identity,” comprised five items such as “I thought that this event brought about a change in my life” and “I thought that this event was a turning point in my life.” Factor 3, named “Facilitating communication,” comprised four items such as “I thought it would deepen my sense of unity with my friends and acquaintances” and “I thought it would create a common connection between old and new friends.” Factor 4, named “Coping with negative emotion,” comprised three items, including “I felt embarrassed” and “I tried to forget this ever happened.” To assess internal consistency, we calculated Cronbach's α for the FAMOS factors, which was 0.83–0.90, indicating good internal consistency.

**Table 1:** The results of factor analysis of the FAMOS.

**Factor**	**1**	**2**	**3**	**4**	**Communality**	***M (SD)***
F1: Evoking positive emotion α = 0.90						
I felt comfortable.	0.94	−0.04	−0.05	0.05	0.80	4.89 (1.83)
I relaxed.	0.81	−0.06	0.09	0.07	0.65	4.40 (1.86)
I felt nostalgic.	0.76	0.03	−0.08	−0.04	0.58	5.58 (1.67)
It was fun.	0.74	−0.01	0.13	−0.01	0.63	4.40 (1.84)
I reconfirmed that I like the smell.	0.73	0.12	−0.07	−0.08	0.58	5.08 (1.91)
F2: Identity α = 0.90						
I thought that this event brought about a change in my life.	−0.04	0.84	0.01	−0.02	0.68	3.65 (1.89)
I thought this event illustrated an important theme in my life.	−0.01	0.83	−0.02	0.02	0.67	3.61 (1.93)
I thought that this event was a turning point in my life.	−0.08	0.78	0.02	0.12	0.66	3.19 (1.86)
I reconfirmed that this event was actually important.	0.09	0.73	0.02	−0.05	0.60	3.93 (1.92)
At the time, I thought this event would mean something to me.	0.10	0.71	0.00	−0.06	0.55	4.09 (1.96)
F3: Facilitating communication α = 0.90						
I thought it would help me feel more at one with my friends and acquaintances.	0.00	−0.02	0.87	−0.04	0.71	3.03 (1.71)
I thought it would create a common connection between old and new friends.	0.04	−0.02	0.84	0.01	0.71	2.94 (1.69)
I shared memories with friends and acquaintances, which deepened my intimacy	0.02	0.05	0.81	0.02	0.72	2.91 (1.67)
in interpersonal relationships.						
I shared memories with friends and acquaintances, which helped me	−0.06	0.04	0.80	0.00	0.64	2.89(1.71)
maintain relationships.						
F4: Coping with negative emotion α = 0.83						
I felt embarrassed.	0.08	−0.05	−0.04	0.88	0.69	2.11 (1.50)
I've tried to forget that this ever happened.	−0.14	−0.01	−0.02	0.76	0.64	2.02 (1.44)
I tried to avoid what I had to do.	0.04	0.09	0.11	0.70	0.63	2.36 (1.51)
Factor correlation	1					
	2	0.41				
	3	0.33	0.57			
	4	−0.27	0.31	0.49		

*The bold values indicate the factor loadings of the items corresponding to each factor*.

Factors 1 and 4 are emotion-related factors and correspond to the emotional regulation functions suggested by previous studies (e.g., Erber and Erber, 1994; Smith and Petty, 1995). As already mentioned, odor has a strong emotion-arousing power, which may be interpreted as promoting the generation of emotional functions. Factor 2 is related to the persistence and consistency of the self, which corresponds to the self function in previous studies (Bluck, 2003; Bluck et al., 2005). Factor 3 is related to the sustainability of conversations and relationships with others, which corresponds to the social function in previous studies (Bluck, 2003; Bluck et al., 2005). Additionally, Yamamoto et al.'s (2018b) study, which asked participants to freely describe their behavior and thoughts after autobiographical memories were evoked by taste and odor using Shikohin (e.g., alcohol, tea, and coffee) cues, confirmed that odors could facilitate communication, which may be a unique result of olfactory stimulation. Moreover, Factor 4 was confirmed as the function of autobiographical memories evoked by the consumption of Shikohin products (Yamamoto and Yokomitsu, 2019). Overall, two of the three conventional theoretical functions were reproduced as functions of autobiographical memory cued by odor. The unreproducible directive function was interpreted to be further separated into two emotional regulation functions by the unique emotion-arousing power of odor.

## Study 2

### Purpose

To examine the concomitant validity of the FAMOS, we used several measures and scales. First, we adopted ratings for the emotional characteristics of odors and had participants rate the emotional arousal and valence of the odor cues. The higher the emotional characteristics of the odor cue, the higher the characteristics of the recalled autobiographical memory (Yamamoto and Sugiyama, 2017). Therefore, the emotional characteristics of odor cues are expected to affect the memory functions evoked by odors. Second, we used the OEAMQ, which measures the memory properties recalled by odor cues. The higher the recalled memory property, the more likely it is that the functions are facilitated. Third, we used the olfactory image vividness questionnaire (VOIQ) (Gilbert et al., 1998). High olfactory imagery ability in individuals affects memory performance (Willander and Larsson, 2008), which implies that higher olfactory imagery facilitates function. Fourth, the Odor Awareness Scale (OAS) (Smeets et al., 2008) was used to measure the degree of interest in scent. It is predicted that the higher the level of interest in the scent, the more the function of the memory recalled by the smell is facilitated. Thus, in Study 2, the validity of the FAMOS was examined using these ratings and scales.

### Methods

#### Participants

Study 2's participants were 600 healthy older people in Japan (300 female participants, mean age = 70.21 years, SD = 4.56, range 65–89 years). The criteria and treatment were the same as in Study 1. None of the participants in Study 2 participated in Study 1.

#### Questionnaire

##### Odor emotional ratings

Similar to Yamamoto and Sugiyama (2017), the following two items pertaining to the emotional cognitive aspects of odors were rated on a 7-point scale: emotional arousal (“How much emotion was aroused by smelling the odor?”; 1 = not at all, 7 = very much) and emotional valence (“How positive or negative is that emotion?”; 1 = very negative, 7 = very positive).

##### Odor-evoked autobiographical memory questionnaire

Excluding the factors used in Study 1, 12 items related to emotion, clarity, time information, and perceptual experience were used. One item of the emotion factor was used in Study 1, but it was not adopted in the FAMOS; therefore, it was used in this study.

##### Vividness odor of imagery questionnaire

This scale, originally developed by Gilbert et al. (1998), measures an individual's olfactory imagery. The Japanese version of the VOIQ has a one-factor structure, and its reliability and validity have been confirmed (Yamamoto et al., 2018a). This scale comprised 14 items graded on a 5-point scale.

##### Odor awareness scale

This scale, developed by Smeets et al. (2008), measures individual differences in the perceived ease of awareness of odors. The Japanese version of the OAS was developed by Nakano and Ayabe-Kanamura (2014). This version comprises 20 items graded on a 4-point scale.

### Procedure

The same online diary method as in Study 1 was used in Study 2. Participants recorded their memory recall experiences and then responded to the emotional characteristics of the odor cues using the questionnaire.

### Results and discussion

Four participants with missing values were excluded. As a result, 596 participants were included in the analysis. To assess the validity of the FAMOS, a correlational analysis of the relationship between the FAMOS factors and each scale was performed. As shown in Table 2, some significant correlations were found between the FAMOS factors and some scales. First, it was confirmed that the more positive the emotional characteristics of the odor were, the higher the scores tended to be for the first three factors: Evoking positive emotions, Identity, and Facilitating communication. Factor 4 showed a significant negative correlation only with valence; the more positive emotions were aroused, the better respondents tended to cope with negative emotions. The same tendency was observed in the OEAMQ, especially in the emotion factor, suggesting that the emotional characteristics of recalled memories are related to each factor of the FAMOS. No significant correlation coefficients were found between the FAMOS, and the VOIQ and OAS. These results suggest that the FAMOS may not be related to individual differences in odor awareness and olfactory imagery ability. Although some of the results were not significant, overall, the results suggest a fair degree of validity for the FAMOS.

**Table 2:** The correlation coefficient between each variable.

**Scale and ratings**	**FAMOS F1**	**FAMOS F2**	**FAMOS F3**	**FAMOS F4**	***M (SD)***
Emotional arousal	−0.02	0.09	−0.04	−0.01	3.79 (0.77)
Emotional valence	0.75**	0.23**	0.31**	−0.32**	3.70 (1.32)
OEAMQ emotion	0.86**	0.32**	0.37**	−0.39**	14.96 (5.46)
OEAMQ clarity	0.09	0.27**	0.06	−0.07	17.12 (3.64)
OEAMQ time information	0.10	0.28**	0.11**	−0.00	16.79 (4.17)
OEAMQ perceptual experience	0.43**	0.37**	0.33**	−0.08	14.82 (3.88)
VOIQ	0.06	0.13**	0.06	−0.06	50.71 (11.43)
OAS	0.10	0.17**	0.05	−0.01	74.43 (11.00)
M (SD)	23.05 (9.26)	20.38 (8.10)	13.52 (7.03)	7.42 (4.14)	

*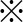 p < 0.01^**^*.

## Study 3

### Purpose

To examine the discriminant validity of the FAMOS, we examined the generational differences between younger and older adults. Recently, it has been reported that there are generational differences in functions of autobiographical memory (e.g., Bluck and Alea, 2009; Alea et al., 2015; Vranić et al., 2018). It is important to consider generational differences given that autobiographical memory covers an entire lifetime. For example, Bluck and Alea (2009) reported that younger people used the self and directive functions more frequently than older people, but no generational differences were found in the social function. Vranić et al. (2018) also reported that younger people tend to use the directive function more than older people, while no age differences were found in the use of the self function. In addition, cultural factors have been shown to influence generational differences in functions of autobiographical memory (Alea et al., 2015). As this scale was designed for older adults, it is naturally expected that these values would be higher in older adults than in younger adults. Thus, in Study 3, generational differences between younger and older adults were examined in a validity study for FAMOS.

### Methods

#### Participants

Study 3's participants were recruited using the same criteria as in Studies 1 and 2, except that they were aged 20–29 years. The treatment was the same as in Studies 1 and 2. The participants were 600 healthy young people in Japan (300 female participants, mean age = 25.29 years, SD = 2.69, range 20–29 years). Data from Study 2 were used for the older adults.

#### Procedure

The same online diary method used in Studies 1 and 2 was used for Study 3. Participants recorded their memory recall experiences and then responded to the FAMOS.

### Results and discussion

The total mean per factor was calculated for the collected data, and an independent sample *t-*test was conducted to compare the results with the older adult data from Study 2. The results of the analysis are shown in Table 3. The older group had higher scores for Factor 1 (Evoking positive emotion), Factor 2 (Identity), and Factor 3 (Facilitating communication) than the younger group. Factor 4 (Coping with negative emotion) showed no difference between the groups.

**Table 3:** Means and *t*-test results for each the FAMOS factor in each group.

**FAMOS factor**	**Younger**	**Older**	***t*** **(*****df*** = **1.194)**	***d***
F1: Evoking positive emotion	21.48 (7.84)	23.05 (9.26)	3.17*	0.18
F2: Identity	16.82 (7.89)	20.38 (8.10)	7.71**	0.45
F3: Facilitating communication	11.69 (6.33)	13.52 (7.03)	4.71**	0.27
F4: Coping with negative emotion	7.78 (4.32)	7.42 (4.14)	1.45	0.08

*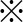 p < 0.005^*^, p < 0.001^**^, SD in parentheses*.

Generational differences were found in the first factors related to emotion. In recent years, autobiographical memory research has pointed to the age-related positivity effect (e.g., Kennedy et al., 2004; Gallo et al., 2011). This effect refers to older adults tending to remember the events of their lives more positively than younger adults. When asked to generate autobiographical memories in response to retrieval cues, the number of positive events tends to increase with age compared to negative events (Dijkstra and Kaup, 2005; Ros and Latorre, 2010). Older adults also tend to rate retrieved events more positively than younger adults (Rubin and Schulkind, 1997; Comblain et al., 2005). The effect has been explained by the socio-emotional selectivity theory (SST) (see review, Carstensen et al., 2006). According to SST, aging is associated with a perception of shrinking time horizons (i.e., limited remaining lifespan), and this perception motivates people to shift their goals toward optimizing emotional regulation and focusing on the more positive aspects of experience (Mather and Carstensen, 2005). Therefore, older adults recall more positive memories than negative ones compared to younger adults. Even among younger adults, many autobiographical memory studies have demonstrated preferences for positive events over negative events (e.g., Walker et al., 2003; Wilson and Ross, 2003; Fotopoulou et al., 2007), potentially owing to the biasing effects of a positive self-concept (Conway, 2005).

The age-related positivity effect has also been found when participants involuntarily remember autobiographical memories during the course of the day (e.g., Schlagman et al., 2006) and when participants explicitly try to remember self-evaluations that they had previously recorded in surveys or diaries (Levine and Bluck, 1997; Kennedy et al., 2004; Ready et al., 2007). This effect may have been generated in this study as well. Therefore, the values related to the positive emotion arousal function were lower in older adults than in younger adults because positive emotions were preferentially aroused in older adults.

Generational differences were also observed for Factor 2 (Identity). The results of this study are different from those of previous studies, including Bluck and Alea's (2009) study, which found that younger people use self-functioning more than older people. Factor 2, however, includes many items such as “I thought that this event was a turning point in my life” and “I thought this event illustrated an important theme in my life,” which capture the entirety of one's life. This explains why the values of these factors were higher in older participants than in younger participants.

Furthermore, generational differences were observed for Factor 3 (Facilitating communication), suggesting that older adults recall memories more often than younger adults to facilitate communication. Our results contrast the results of Bluck and Alea (2009), where no generational difference was found for social function. Furthermore, Alea et al. (2015) suggested that young adults think and talk about autobiographical memories for social bonding. These differences in findings may be a result of the questionnaire used in this study; the scale was specifically designed for older adults and provided them with an advantage in the third factor (e.g., “I thought it would create a common connection between old and new friends.”). Therefore, older participants scored higher on Factor 3 than younger participants in the present study. Moreover, older adults possess more autobiographical memories than younger adults, and thus have a larger pool of memories available for recall. Thus, autobiographical memory is often used effectively in interpersonal situations.

Additionally, no generational difference was found for Factor 4 (Coping with negative emotion). According to Mather (2016), aging is a multifaceted process that involves interaction between brain regions and neurotransmitter systems, which are not uniformly affected by aging. Although aging is associated with apparent declines in physical and cognitive processes, emotional functioning fares relatively well. Consistent with this behavioral profile, two core emotional brain regions—the amygdala and ventromedial prefrontal cortex—may show minor structural and functional decline upon aging compared with other areas (Mather, 2016). Thus, it is possible that generational differences may not have occurred in coping with negative emotion.

## Conclusion

In these three studies, we developed the FAMOS to measure the functions of autobiographical memory evoked by odor among older Japanese people. Consequently, a four-factor scale was proposed, which showed a fair degree of reliability and validity. In the following section, the limitations of this study and future research directions are discussed.

One limitation is that the data were obtained only from Japanese participants. Previous studies have pointed out the possibility that culture may have an impact (Alea et al., 2015). To further confirm the reliability of the FAMOS, it is necessary to examine the data of participants from other cultures. Another issue is the decline in cognitive function among older adults. Several reports have shown that decreased olfaction is common among older people (for a review, see Attems et al., 2015). Previous studies have indicated that this presents in >50% of individuals aged between 65 and 80 years and in 62–80% of those over 80 years of age (Attems et al., 2015). Smell dysfunction significantly influences physical wellbeing, quality of life, nutritional status, and everyday safety and is associated with increased mortality. This study included healthy older adults who self-reported having no problems with their sense of smell, but their sense of smell was not objectively measured. This issue should be addressed in future studies. In addition, although the diary method was used in the present study, other research methods have been developed for researching involuntary memory (for a review of methodology, see Berntsen, 2021). It is important to study the reliability of the FAMOS by examining whether similar results are obtained using other methods.

Finally, the possibilities for future research applications are reviewed. As mentioned in Section Introduction, the recall of autobiographical memories by means of odor cues is expected to be linked to the mental health of older adults. The results of the FAMOS inter-factor correlations (see Table 1) confirm moderate correlation, which suggests that the recall of autobiographical memories by odor cues may facilitate the development of identity and communication, and that these may also influence the evocation of positive emotions. These factors may interactively influence each other and enhance older adults' subjective wellbeing, which may be explored in future research. Patients with Alzheimer's disease also showed better specificity, emotional experience, mental time travel, and retrieval time after odor and music exposure than those in the control condition (e.g., El Haj et al., 2018). Similar beneficial effects of odor and music exposure were observed for autobiographical characteristics, except for retrieval time, which improved more after odor exposure than after music exposure. The FAMOS developed in this study is expected to influence the sense of wellbeing of patients with Alzheimer's disease by arousing positive emotions. In the future, we would like to investigate the possibility of applying the FAMOS to a wide range of subjects, not only to healthy older adults. Furthermore, it is possible that FAMOS could be used to assess autobiographical memories recalled by not only odors but also other sensory-perceptual cues (e.g., musical cues, as noted by El Haj et al., 2013). Further research is needed to investigate the applicability of the FAMOS to other cues.

## Data availability statement

The raw data supporting the conclusions of this article will be made available by the authors, without undue reservation.

## Ethics statement

The studies involving human participants were reviewed and approved by Local Research Committee of Osaka Sangyo University. The patients/participants provided their written informed consent to participate in this study.

## Author contributions

KYa, KYo, and TK: conceptualization and investigation. Kya and KYo: data curation. Kya: formal analysis. Kya and TK: funding acquisition. KYo: project administration and writing–original draft. KYo and TK: writing–review and editing. All authors contributed to the article and approved the submitted version.

## Funding

This work was funded by Grants-in-Aid for Scientific Research (KAKENHI), Japan Society for the Promotion of Science (JSPS), Grant Nos. 17K13924, 20H01223, and 22K03092.

## Conflict of interest

The authors declare that the research was conducted in the absence of any commercial or financial relationships that could be construed as a potential conflict of interest.

## Publisher's note

All claims expressed in this article are solely those of the authors and do not necessarily represent those of their affiliated organizations, or those of the publisher, the editors and the reviewers. Any product that may be evaluated in this article, or claim that may be made by its manufacturer, is not guaranteed or endorsed by the publisher.

## References

[B1] Alea N. Bluck S. Ali S. (2015). Function in context: why American and Trinidadian young and older adults remember the personal past. Memory. 23, 55–68. 10.1080/09658211.2014.929704 24992649 PMC4282835

[B2] Arshamian A. Iannilli E. Gerber J. C. Willander J. Persson J. Seo H. S.. (2013). The functional neuroanatomy of odor evoked autobiographical memories cued by odors and words. Neuropsychologia. 51, 123–131. 10.1016/j.neuropsychologia.2012.10.023 23147501

[B3] Attems J. Walker L. Jellinger K. A. (2015). Olfaction and aging: a mini-review. Gerontology. 61, 485–490. 10.1159/000381619 25968962

[B4] Berntsen D.. (1996). Involuntary autobiographical memories. Appl. Cognit. Psychol. 10, 435–454.

[B5] Berntsen D.. (2021). Involuntary autobiographical memories and their relation to other forms of spontaneous thoughts. Phil. Trans. R. Soc. B. 376, 20190693. 10.1098/rstb.2019.0693 33308074 PMC7741080

[B6] Berntsen D. Hall N. M. (2004). The episodic nature of involuntary autobiographical memories. Mem. Cognit. 32, 789–803. 10.3758/BF03195869 15552356

[B7] Bluck S.. (2003). Autobiographical memory: exploring its functions in everyday life. Memory. 11, 113–123. 10.1080/741938206 12820825

[B8] Bluck S. Alea N. (2009). Thinking and talking about the past: why remember? Appl. Cognit. Psychol. 23, 1089–1104. 10.1002/acp.161216519004

[B9] Bluck S. Alea N. (2011). Crafting the TALE: construction of a measure to assess the functions of autobiographical remembering. Memory. 19, 470–486. 10.1080/09658211.2011.590500 21864212

[B10] Bluck S. Alea N. Habermas T. Rubin D. C. (2005). A tale of three functions: the self-reported uses of autobiographical memory. Soc. Cogn. 23, 91–117. 10.1521/soco.23.1.91.5919826371517

[B11] Carstensen L. L. Mikels J. A. Mather M. (2006). “Aging and the intersection of cognition, motivation and emotion,” in Handbook of the Psychology of Aging, 6th ed., eds J. E. Birren, and K. W. Schaie (San Diego, CA: Academic Press), 343–362.

[B12] Chu S. Downes J. J. (2000a). Odour-evoked autobiographical memories: psychological investigations of Proustian phenomena. Chem. Senses. 25, 111–116. 10.1093/chemse/25.1.111 10668001

[B13] Chu S. Downes J. J. (2000b). Long live proust: the odour-cued autobiographical memory bump. Cognition. 75, B41–B50. 10.1016/S0010-0277(00)00065-2 10771279

[B14] Chu S. Downes J. J. (2002). Proust nose best: odors are better cues of autobiographical memory. Mem. Cognit. 30, 511–518. 10.3758/BF03194952 12184552

[B15] Comblain C. D'Argembeau A. Van der Linden M. (2005). Phenomenal characteristics of autobiographical memories for emotional and neutral events in older and younger adults. Exp. Aging Res. 31, 173–189. 10.1080/03610730590915010 15981795

[B16] Conway M. A.. (2005). Memory and the self. J. Mem. Lang. 53, 594–628. 10.1016/j.jml.2005.08.005

[B17] Dijkstra K. Kaup B. (2005). Mechanisms of autobiographical memory retrieval in younger and older adults. Mem. Cognit. 33, 811–820. 10.3758/BF03193076 16383169

[B18] El Haj M.. (2022). Odor-evoked autobiographical memory in Alzheimer's disease? Arch. Clin. Neuropsychol. 37, 513–520. 10.1093/arclin/acab074 34491308

[B19] El Haj M. Clément S. Fasotti L. Allain P. (2013). Effects of music on autobiographical verbal narration in Alzheimer's disease. J. Neurolinguistics. 26, 691–700. 10.1016/j.jneuroling.2013.06.001

[B20] El Haj M. Gandolphe M. C. Gallouj K. Kapogiannis D. Antoine P. (2018). From nose to memory: the involuntary nature of odor-evoked autobiographical memories in Alzheimer's disease. Chem. Senses. 43, 27–34. 10.1093/chemse/bjx064 29040475 PMC5863564

[B21] Engen T.. (1982). The Perception of Odors. New York, NY: Academic Press.

[B22] Erber R. Erber M. W. (1994). Beyond mood and social judgment: mood incongruent recall and mood regulation. Eur. J. Soc. Psychol. 24, 79–88. 10.1002/ejsp.2420240106

[B23] Fotopoulou A. Conway M. A. Solms M. (2007). Confabulation: motivated reality monitoring. Neuropsychologia. 45, 2180–2190. 10.1016/j.neuropsychologia.2007.03.003 17428509

[B24] Gallo D. A. Korthauer L. E. McDonough I. M. Teshale S. Johnson E. L. (2011). Age-related positivity effects and autobiographical memory detail: evidence from a past/future source memory task. Memory. 19, 641–652. 10.1080/09658211.2011.595723 21919591 PMC3623554

[B25] Gilbert A. N. Crouch M. Kemp S. E. (1998). Olfactory and visual mental imagery. J. Ment. Imagery. 22, 137–146.

[B26] Habermas T. Bluck S. (2000). Getting a life: the emergence of the life story in adolescence. Psychol. Bull. 126, 748–769. 10.1037/0033-2909.126.5.748 10989622

[B27] Hackländer R. P. M. Janssen S. M. J. Bermeitinger C. (2019). An in-depth review of the methods, findings, and theories associated with odor-evoked autobiographical memory. Psychon. Bull. Rev. 26, 401–429. 10.3758/s13423-018-1545-3 30406397

[B28] Herz R. S.. (2002). “Influences of odors on mood and affective cognition,” in Olfaction, Taste, and Cognition, eds C. Rouby, B. Schaal, D. Dubois, R. Gervais, and A. Holly (New York, NY: Cambridge University Press), 160–177.

[B29] Herz R. S.. (2004). A naturalistic analysis of autobiographical memories triggered by olfactory visual and auditory stimuli. Chem. Senses. 29, 217–224. 10.1093/chemse/bjh025 15047596

[B30] Herz R. S. Cupchik G. C. (1992). An experimental characterization of odor-evoked memories in humans. Chem. Senses. 17, 519–528. 10.1093/chemse/17.5.519 8564426

[B31] Herz R. S. Schooler J. W. (2002). A naturalistic study of autobiographical memories evoked by olfactory and visual cues: testing the proustian hypothesis. Am. J. Psychol. 115, 21–32. 10.2307/1423672 11868193

[B32] Kennedy Q. Mather M. Carstensen L. L. (2004). The role of motivation in the age-related positivity effect in autobiographical memory. Psychol. Sci. 15, 208–214. 10.1111/j.0956-7976.2004.01503011.x 15016294

[B33] Larsson M. Willander J. (2009). Autobiographical odor memory. Ann. N. Y. Acad. Sci. 1170, 318–323. 10.1111/j.1749-6632.2009.03934.x 19686154

[B34] Larsson M. Willander J. Karlsson K. Arshamian A. (2014). Olfactory LOVER: behavioral and neural correlates of autobiographical odor memory. Front. Psychol. 5, 312. 10.3389/fpsyg.2014.00312 24782810 PMC3990043

[B35] Levine L. J. Bluck S. (1997). Experienced and remembered emotional intensity in older adults. Psychol. Aging. 12, 514–523. 10.1037/0882-7974.12.3.514 9308098

[B36] Mather M.. (2016). The affective neuroscience of aging. Annu. Rev. Psychol. 67, 213–238. 10.1146/annurev-psych-122414-033540 26436717 PMC5780182

[B37] Mather M. Carstensen L. L. (2005). Aging and motivated cognition: the positivity effect in attention and memory. Trends Cogn. Sci. 9, 496–502. 10.1016/j.tics.2005.08.005 16154382

[B38] Maylor E. A. Carter S. M. Hallett E. L. (2002). Preserved olfactory cuing of autobiographical memories in old age. J. Gerontol. B Psychol. Sci. Soc. Sci. 57, P41–P46. 10.1093/geronb/57.1.P41 11773222

[B39] Nakano S. Ayabe-Kanamura S. (2014). The applicability of the odor awareness scale to Japanese. Tsukuba Psychol. Res. 47, 1–8.

[B40] Ochiai T. Oguchi T. (2013). Development of a Japanese version of the TALE Scale. Jpn. J. Psychol. 84, 508–514. 10.4992/jjpsy.84.508 24505977

[B41] Pillemer D. B.. (2009). Twenty years after Baddeley (1988): is the study of autobiographical memory fully functional? Appl. Cognit. Psychol. 23, 1193–1208. 10.1002/acp.1619

[B42] Proust M.. (1960). Swann's Way. Translator C. K. S. Moncrieff (London: Chatto & Windus) (original work published 1922).

[B43] Ready R. E. Weinberger M. I. Jones K. M. (2007). How happy have you felt lately? two diary studies of emotion recall in older and younger adults. Cogn. Emot. 21, 728–757. 10.1080/02699930600948269

[B44] Ros L. Latorre J. M. (2010). Gender and age differences in the recall of affective autobiographical memories using the autobiographical memory test. Pers. Individ. Dif. 49, 950–954. 10.1016/j.paid.2010.08.002

[B45] Rubin D. C. Schulkind M. D. (1997). The distribution of autobiographical memories across the lifespan. Mem. Cognit. 25, 859–866. 10.3758/BF03211330 9421572

[B46] Saive A. L. Royet J. P. Plailly J. (2014). A review on the neural bases of episodic odor memory: from laboratory-based to autobiographical approaches. Front. Behav. Neurosci. 8, 240. 10.3389/fnbeh.2014.00240 25071494 PMC4083449

[B47] Sato K.. (2015). Autobiographical reasoning to memories of teachers and families: effects of the motivational orientation towards the teaching profession and the generation. Annual reports of the faculty of education, Gunma University. Cult. Sci. Series 64, 145–156.

[B48] Schlagman S. Schulz J. Kvavilashvili L. (2006). A content analysis of involuntary autobiographical memories: examining the positivity effect in old age. Memory. 14, 161–175. 10.1080/09658210544000024 16484107

[B49] Smeets M. A. M. Schifferstein H. N. J. Boelema S. R. Lensvelt-Mulders G. (2008). The odor awareness scale: a new scale for measuring positive and negative odor awareness. Chem. Senses. 33, 725–734. 10.1093/chemse/bjn038 18622009

[B50] Smith S. M. Petty R. E. (1995). Personality moderators of mood congruency effects on cognition: the role of self-esteem and negative mood regulation. J. Pers. Soc. Psychol. 68, 1092–1107. 10.1037/0022-3514.68.6.1092 7608856

[B51] Takigawa S. Yokomitsu K. (2019). Characteristics differences in reminiscence functions across age groups and types of “shikohin”(luxury or indulgent products). The Jpn. J. Cogn. Psychol. 17, 49–58. 10.5265/jcogpsy.17.49

[B52] Vranić A. Jelić M. Tonković M. (2018). Functions of autobiographical memory in younger and older adults. Front. Psychol. 9, 219. 10.3389/fpsyg.2018.00219 29599732 PMC5863506

[B53] Walker W. R. Skowronski J. J. Thompson C. P. (2003). Life is pleasant – and memory helps to keep it that way! *Rev. Gen. Psychol*. 7, 203–210. 10.1037/1089-2680.7.2.203

[B54] Webster J. D.. (1993). Construction and validation of the reminiscence functions scale. J. Gerontol. 48, P256–P262. 10.1093/geronj/48.5.P256 8366271

[B55] Webster J. D.. (1997). The reminiscence functions scale: a replication. Int. J. Aging Hum. Dev. 44, 137–148. 10.2190/AD4D-813D-F5XN-W07G 9169316

[B56] Willander J. Larsson M. (2006). Smell your way back to childhood: autobiographical odor memory. Psychon. Bull. Rev. 13, 240–244. 10.3758/BF03193837 16892988

[B57] Willander J. Larsson M. (2007). Olfaction and emotion: the case of autobiographical memory. Mem. Cognit. 35, 1659–1663. 10.3758/BF03193499 18062543

[B58] Willander J. Larsson M. (2008). The mind's nose and autobiographical odor memory. Chem. Percept. 1, 210–215. 10.1007/s12078-008-9026-0

[B59] Wilson A. E. Ross M. (2003). The identity function of autobiographical memory: time is on our side. Memory. 11, 137–149. 10.1080/741938210 12820827

[B60] Yamamoto K. Inomata K. Susami K. Ayabe-Kanamura S. (2018a). Development of the Japanese version of the vividness of odor imagery questionnaire. Jpn. J. Pers. 27, 87–89. 10.2132/personality.27.1.10

[B61] Yamamoto K. Inomata K. Tomitaka T. (2016). The functions of involuntary autobiographical memory cued by odor. Jpn J. Taste Smell Res. 23, 115–123. 10.18965/tasteandsmell.23.2_115

[B62] Yamamoto K. Sugiyama H. (2017). Development of the odor-evoked autobiographical memory questionnaire. Jpn. J. Psychol. 77, 478–487. 10.4992/jjpsy.88.16229

[B63] Yamamoto K. Yokomitsu K. (2019). Development of the functions of the autobiographical memory in Shikohin Inventory. Jpn. J. Pers. 28, 67–79. 10.2132/personality.28.1.9

[B64] Yamamoto K. Yokomitsu K. Hirai H. (2018b). The characteristics and functions of involuntary autobiographical memory cued by consuming Shikohin. Jpn. J. Cog. 15, 39–51. 10.5265/jcogpsy.15.39

